# Radiotherapy increases plasma levels of tumoral cell-free DNA in non-small cell lung cancer patients

**DOI:** 10.18632/oncotarget.25053

**Published:** 2018-04-10

**Authors:** Shun-ichiro Kageyama, Keiji Nihei, Katsuyuki Karasawa, Takeshi Sawada, Fumiaki Koizumi, Shigeo Yamaguchi, Shunsuke Kato, Hidehiro Hojo, Atsuhi Motegi, Katsuya Tsuchihara, Tetsuo Akimoto

**Affiliations:** ^1^ National Cancer Center Hospital East, Kashiwa, Chiba 277-8577, Japan

**Keywords:** tumoral cell-free DNA, radiotherapy, NSCLC, tumor-specific mutations, digital PCR

## Abstract

We investigated the plasma levels of tumor-specific cell-free DNA (cfDNA) in 17 stage I–II (early) and IV (advanced) non-small cell lung cancer (NSCLC) patients who underwent radiotherapy. Digital polymerase chain reaction (PCR) and targeted sequencing showed that total and tumor-specific cfDNA levels increased in response to radiotherapy in both early- and advanced-stage NSCLC patients. We detected high copy numbers of epidermal growth factor receptor mutations (L858R and T790M) in the cfDNA samples from stage IV NSCLC patients who underwent stereotactic body radiation therapy to treat brain metastasis related to tyrosine kinase inhibitor (TKI) treatment failure. In conclusion, our study demonstrates that radiotherapy increases tumoral cfDNA levels in the plasma and shows potential to serve as an indicator for diagnosing drug-resistant tumor-related gene mutations in early-stage NSCLC patients or those undergoing molecular targeted therapy.

## INTRODUCTION

Analysis of tumoral cell-free DNA (cfDNA) represents a cost-effective, non-invasive method to detect tumor-related gene mutations and drug resistance in human cancers; higher degree tumor cell necrosis is associated with higher plasma levels of cfDNA [1–6]. Diehl *et al.* showed that the majority of the tumoral cfDNA fragments represent nucleosomal units (160 to 200 base pairs long) [7, 8, 12], with half-lives ranging from a few minutes to several hours [10, 13]. These fragments are cleared in the spleen, liver, and kidneys [9–11], thought the specific cfDNA clearance mechanisms are not well understood.

CfDNA analysis has been used to assess drug resistance in non-small cell lung cancer (NSCLC) [15]. A phase III clinical trial (NCT02282267) used digital polymerase chain reaction (PCR) analysis to quantify dynamic changes in epidermal growth factor receptor (EGFR) mutations in NSCLC patients in patient cfDNA samples. Most studies have used PCR to make diagnoses based on known genetic variations in cfDNA samples, which have a sensitivity of 70–90% and a specificity of 90% [2, 8, 14, 15].

Next-generation sequencing of cfDNA samples from untreated stage IV NSCLC patients has shown promise as a diagnostic tool [16, 17]. However, cfDNA detection is difficult in early-stage cancer patients or cancer patients undergoing therapy. This is critical because nearly 50% of NSCLC patients are diagnosed with early stage disease. Recently, a targeted sequencing approach with 10,000× coverage to detect somatic driver mutations in cfDNA from early-stage NSCLC patients ( IA, IB, and IIA) showed a concordance rate of 39.8% with sensitivity and specificity of 53.8% and 47.3%, respectively [2, 18, 19]. Therefore, there is a need to increase tumoral cfDNA levels in early-stage cancer patients and those undergoing treatment in order to optimally use cfDNA analysis for diagnostic purposes.

Radiotherapy is widely used for cancer treatment and palliative care because targeted irradiation increases tumor cell apoptosis. NSCLC patients undergo stereotactic body radiotherapy (SBRT), chemo radiotherapy, or palliative radiotherapy at different stages of the disease. Since higher levels of cell-free DNA and CTC have been observed in the plasma of patients during chemo radiotherapy [7, 20], we postulated that tumoral cfDNA analysis after radiotherapy would be useful to diagnose cancer-related mutations and chemo resistance in early stage cancer patients and those undergoing treatment.

Radiation doses vary widely among cell lines, animal models, and clinical settings. In a laboratory setting, cancer cell lines are irradiated with 2–20 Gy/fraction (fr) and undergo apoptosis within 24–48 h [21, 22]. In animal experiments, the radiation doses are higher (10 Gy/5 fr to 30 Gy/15 fr) and tumor reduction is observed within 1–2 weeks after irradiation [23–25]. In the clinic, radiation doses vary between 60 Gy/30 fr and 50 Gy/4 fr, and maximal tumor reduction is observed within 4–8 weeks after irradiation [26, 27]. In the present study, we determined the effects of radiotherapy on the levels of tumoral cfDNA in 17 NSCLC patients (stages I and IV).

## RESULTS

**Figure 1:**
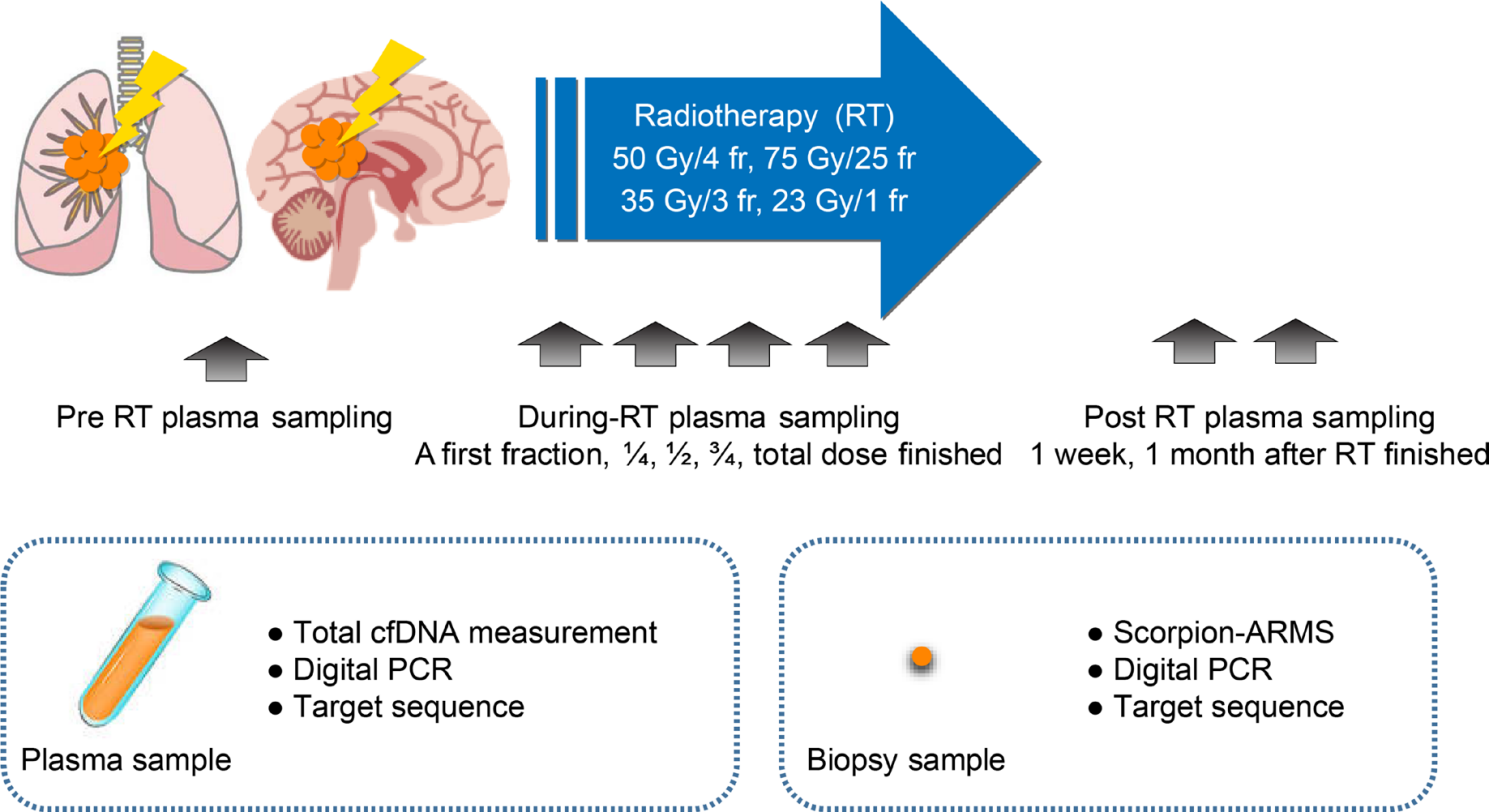
Schematic representation of the experimental strategy to analyze plasma cfDNA levels in NSCLC patients in response to radiotherapy The plasma and biopsy samples were obtained from NSCLC patients during (24 h) and post-radiotherapy (at 1 week and 1 month) and compared with pre-radiotherapy samples. Total cfDNA levels and tumor-specific cfDNA levels were estimated by digital PCR and targeted sequencing. Note: cfDNA, cell-free DNA; fr, fraction; RT, radiotherapy.

We enrolled seventeen patients with suspected NSCLC that underwent curative or palliative radiotherapy between July 2013 and July 2015. Eleven stage I NSCLC patients rejected surgery and chose radiotherapy, whereas, the remaining 6 stage IV NSCLC patients were positive for *EGFR* mutations and showed new brain metastasis, suggesting failure of tyrosine kinase inhibitor (TKI) treatment. The 11 stage I primary NSCLC patients were treated with curative SBRT or 3D-CRT, whereas, the six stage IV NSCLC patients received palliative SBRT against brain metastasis. A high-radiation dose was delivered specifically to the cancer tissue as routine therapy for all patients as shown in Supplementary Figure 1. The experimental strategy for this study is shown in Figure 1. The clinicopathological features of the 17 patients are described in Table 1.

**Table 1:** Clinical characteristics of NSCLC patients

Case	Age/Gender	TNM	Stage	Biopsy	Histology	*EGFR* mutation	Radiation dose
Case 1	70/F	T1bN0M0	IA	−	ad	N.D.	75 Gy/25fr
Case 2	90/M	T2aN0M0	IB	+	ad	L858R	75 Gy/25fr
Case 3	84/M	T1aN0M0	IA	−	ad	N.D.	75 Gy/25fr
Case 4	84/F	T2aN0M0	IB	+	ad	N.D.	75 Gy/25fr
Case 5	73/F	T1aN0M0	IA	−	ad	Wild	50 Gy/4fr
Case 6	91/F	T1aN0M0	IA	−	ad	N.D.	50 Gy/4fr
Case 7	85/M	T2aN0M0	IB	+	ad	N.D.	50 Gy/4fr
Case 8	73/F	T2aN0M0	IB	+	NSCLC	N.D.	50 Gy/4fr
Case 9	67/M	T1aN0M0	IV	−	ad	N.D.	50 Gy/4fr
Case 10	83/M	T1aN0M0	IA	+	ad	N.D.	50 Gy/4fr
Case 11	85/F	T1aN0M0	IA	−	ad	N.D.	45 Gy/4fr
Case 12	63/F	T2bN0M01b (BRA)	IV	+	adsq	exon 19 del.	35 Gy/3fr
Case 13	40/M	T4NXM1b (PUL, OSS, BRA)	IV	+	ad	exon 19 del.	35 Gy/3fr
Case 14	52/M	T1aN3M1 (BRA, PUL)	IV	+	ad	T790M	23 Gy/1fr
Case 15	71/M	T3NXM1b (PUL, BRA, OSS, HEP, SPL)	IV	+	ad	L858R	23 Gy/1fr
Case 16	45/M	T2aN3M1b (OSS, BRA, HEP)	IV	+	ad	L858R	23 Gy/1fr
Case 17	84/F	T3N2M1b (BRA)	IV	+	NSCLC	exon 19 del.	23 Gy/1fr

Note: ad., adenocarcinoma; adsq. adenosquamous; Stage, cancer stages I–IV; fr, fraction; N.D., not determined; NSCLC, non-small cell lung cancer; TNM, classification system of malignant tumors.

Metastasis site: PUL, lung; BRA., brain; HEP., liver; OSS., bone; SPL., spleen.

**Figure 2:**
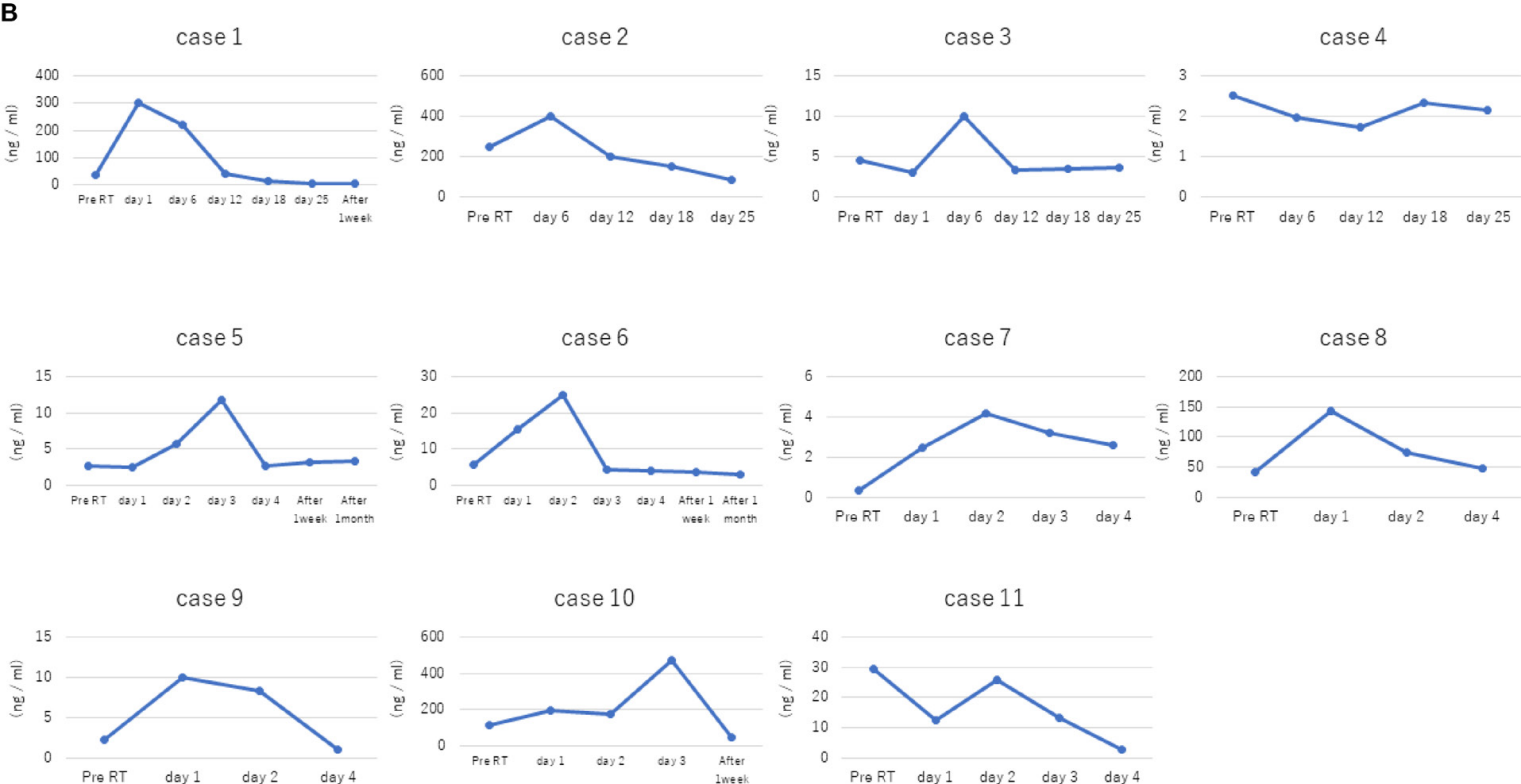

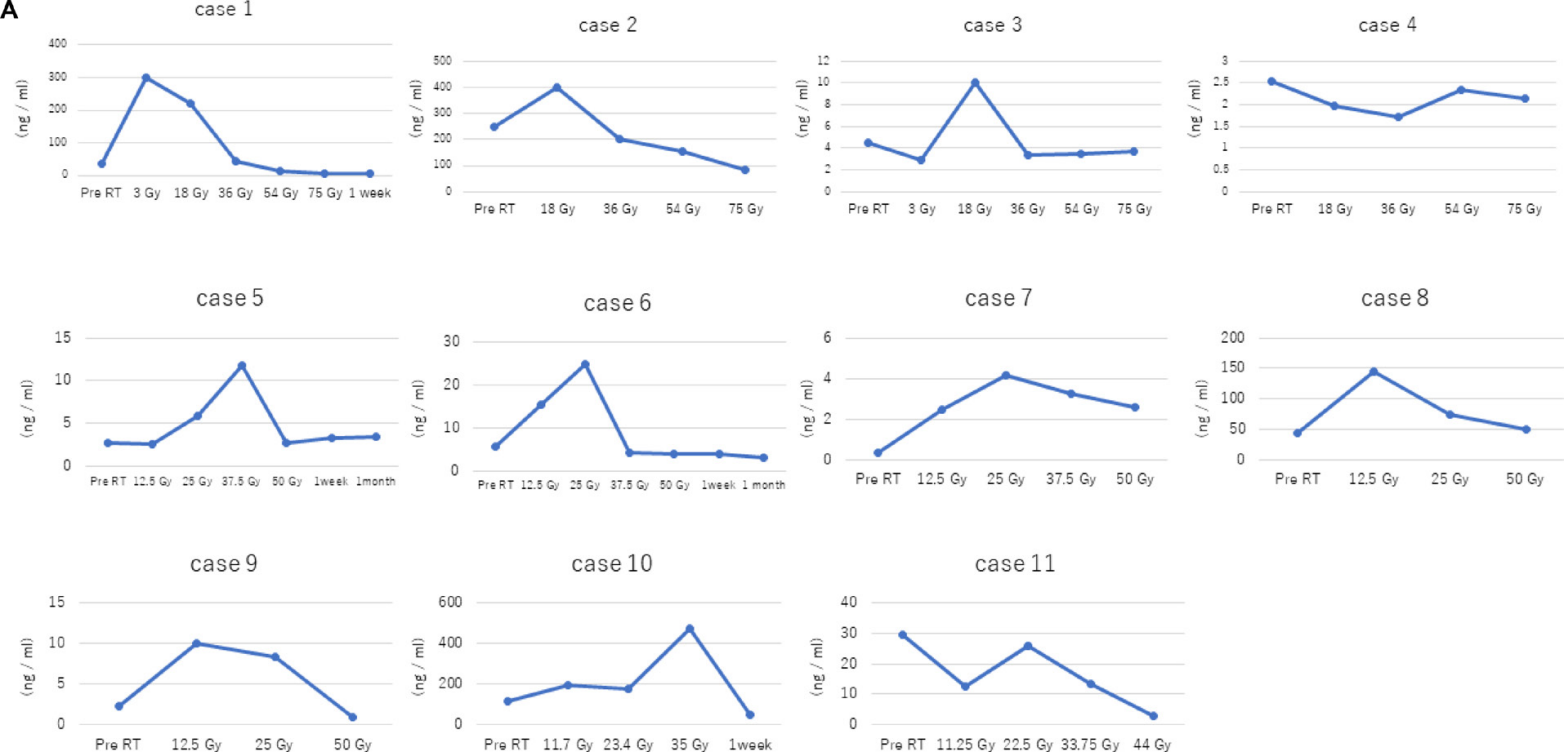
Dose- and time-dependent increase in plasma cfDNA levels in stage I NSCLC patients in response to radiotherapy (**A**) Plasma cfDNA levels in stage I NSCLC patients (cases 1–11) in response to different irradiation doses are shown. In general, total cfDNA levels increase in response to radiotherapy. (**B**) Plots show time course of plasma cfDNA levels in stage I NSCLC patients (cases 1–11) before (pre-RT) and after radiation therapy. As shown, increased total cfDNA levels are observed at 24 h after radiation therapy.

**Figure 3:**
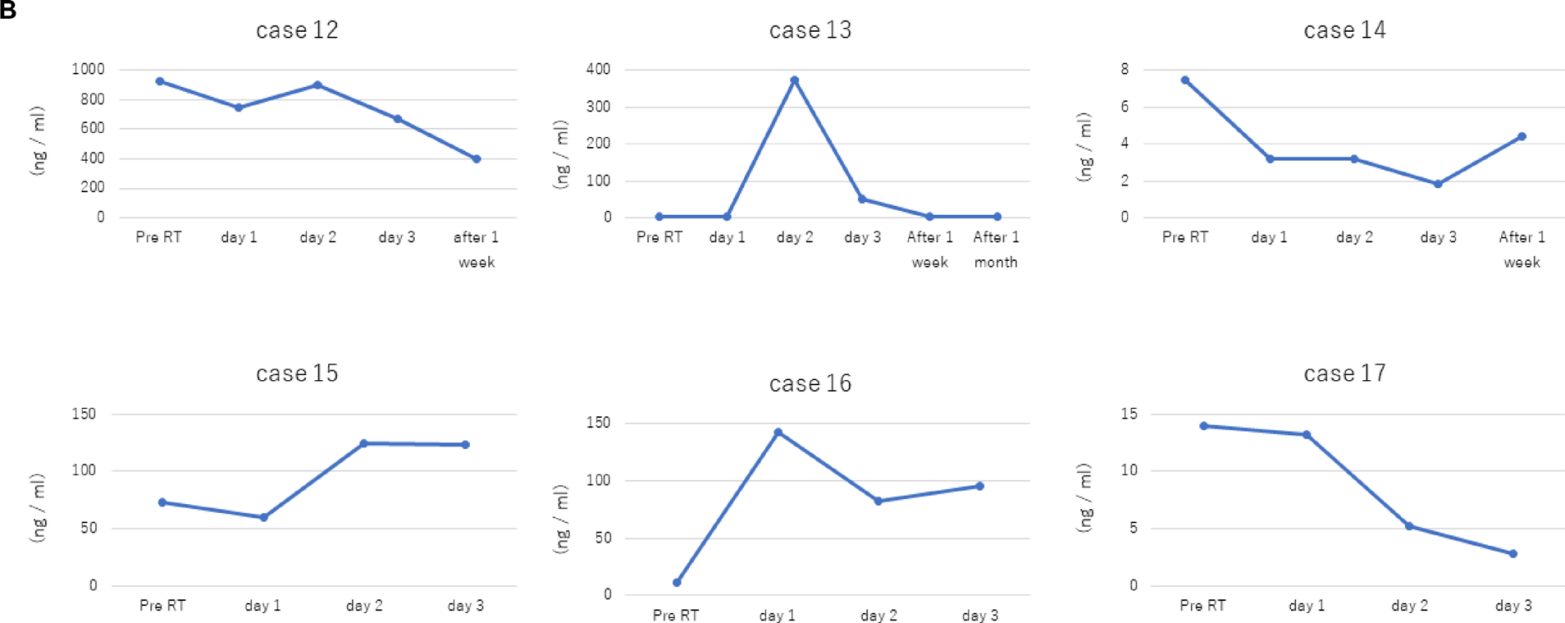

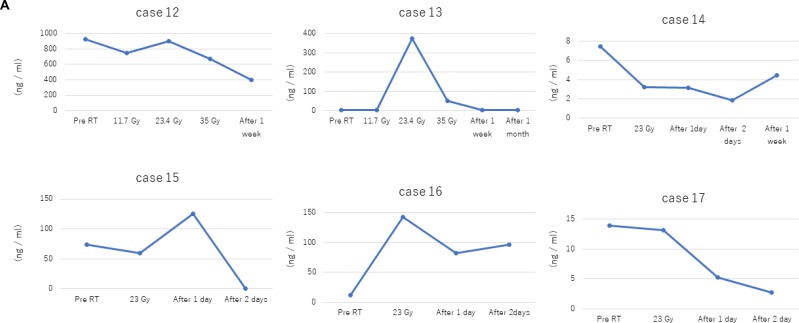
Dose- and time-dependent increase in plasma cfDNA levels in stage IV NSCLC patients in response to radiotherapy (**A**) Plasma cfDNA levels in stage IV NSCLC patients (cases 12–17) in response to different irradiation doses are shown. (**B**) Plots show time course of plasma cfDNA levels in stage IV NSCLC patients (cases 12–17) before (pre-RT) and after radiation therapy.

**Figure 4:**
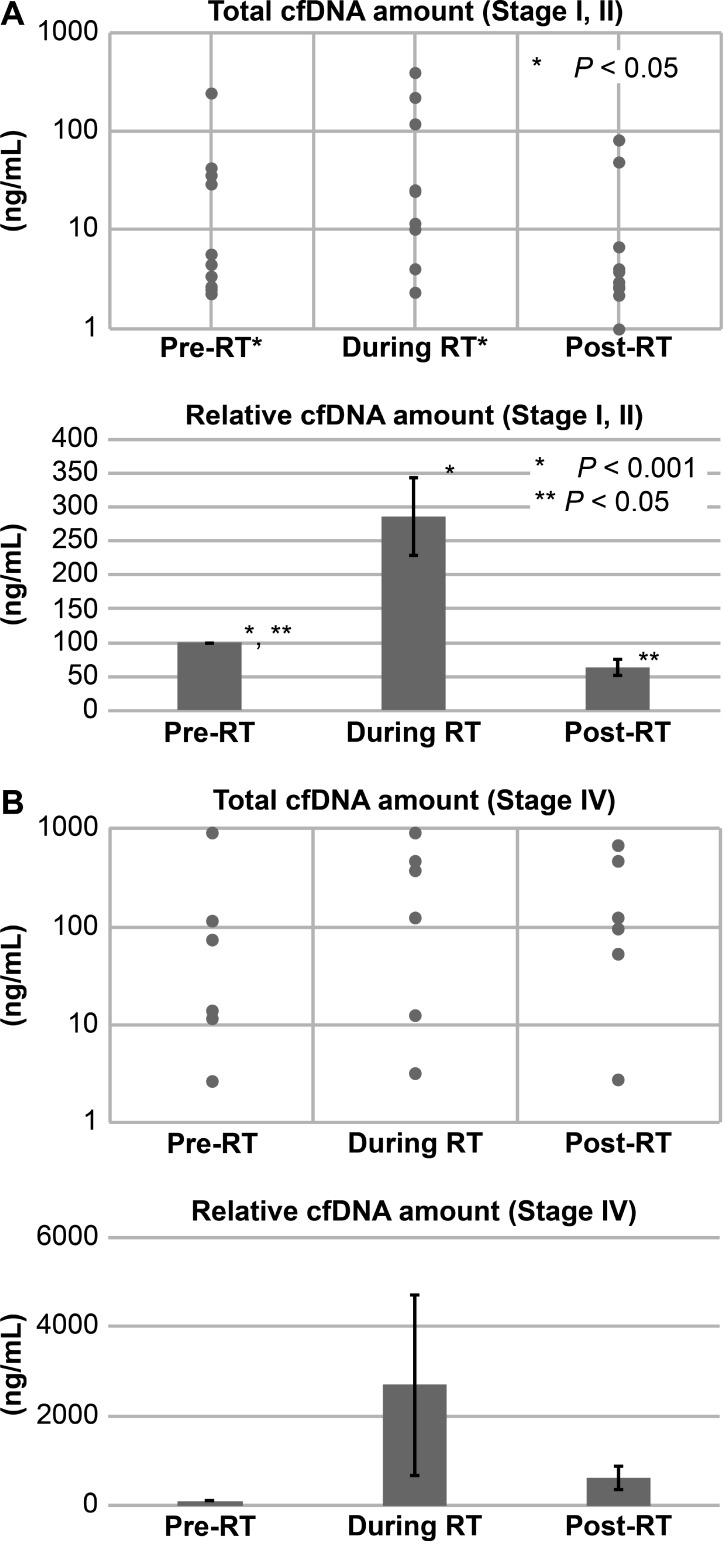
Total and relative cfDNA levels in stage I and IV NSCLC patients before and after radiotherapy Total and relative cfDNA levels in (**A**) stage I–II and (**B**) stage IV NSCLC patients at pre-RT, during RT and post-RT is shown. Note: Continuous variables were compared using Student’s *t*-test and *P* < 0.05 was considered statistically significant compared to pre-RT; RT, radiotherapy.

We detected cfDNA in all patients, and the total circulating cfDNA increased in response to radiotherapy in 12 out of 17 patients (Table 1; Figures 2–3). The median circulating cfDNA concentrations at pre-RT, RT and post-RT in stage I-II NSCLC patients were 5.75, 24.75, and 3.7 ng/mL, respectively (Figure 4A). This demonstrated that the total cfDNA levels increased in response to radiotherapy in stage I NSCLC patients. The median circulating cfDNA concentrations at pre-RT, RT and post-RT in stage IV NSCLC patients were 11.53, 95.0, and 51.83 ng/mL, respectively (Figure 4B). Tumor-specific cfDNA levels were confirmed using digital PCR analysis with matched plasma cfDNA from patients positively diagnosed with EGFR mutation and who provided sufficient DNA for sequence analysis. The relative cfDNA concentration increased significantly between pre-radiotherapy and radiotherapy in stage I–II and stage IV NSCLC patients (*P* < 0.05; Figure 4A–4B). Moreover, post-radiotherapy cfDNA levels were lower than the pre-radiotherapy levels in stage I–II NSCLC patients. The increase in cfDNA levels was detected at 24 h after irradiation and peaked at 7 days after radiotherapy.

Next, we determined the cancer-specific gene mutations in *EGFR*, a dominant driver in lung adenocarcinomas. We performed digital PCR analysis of cfDNA from four patients diagnosed with adenocarcinoma bearing the *EGFR* L858R mutation (*n =* 2) or *EGFR* exon 19 deletion (*n =* 2). The copy numbers for these *EGFR* mutations increased from 0, 20866, 44194, and 64282 copies/mL before radiotherapy to 10562, 139579, 363269, and 2122718 copies/mL, respectively after radiotherapy (Table 2).

**Table 2:** Comparison of allele frequencies of EGFR mutations in cfDNA samples of NSCLC patients as determined by digital PCR and targeted sequencing

Case No	*EGFR* mutation	Allele frequency in pre-RT by digital PCR (copy/mL)	Allele frequency during RT by digital PCR (copy/mL)	Allele frequency in pre-RT by targeted sequencing	Allele frequency during RT by targeted sequencing
case 1	-	-	-	N.D.	7.8% (G719C)
case 2	L858R	N.D (0)	0.9% (10562)	N.D.	0.5%
case 12	exon 19 del.	0.2% (20866)	0.4% (139579)	N.D.	N.D.
case 15	L858R	44194 (0·5%)	2.3%(363269)	0.3%	1.5%
	T790M	N.D. (0)	0.4% (40824)	N.D.	0.3%
case 16	L858R	(N.D.)	0·2%	N.D.	2.8%
	L747S	-	-	N.D.	2.3%
case 17	exon 19 del.	N.D. (64282)	6.4% (2122718)	0.3%	7.3%

Note: del., deletion; N.D., not determined; RT, radiotherapy.

The allele frequencies for the *EGFR* mutations increased from 0, 0.5, 0.2, and 0.6% to 0.9, 2.3, 0.4, and 6.4%, respectively (Table 2). The T790M mutation in case 16 cfDNA sample was detected only after radiotherapy. The diagnosis of case 16 showed lung adenocarcinoma with the L858R mutation and received SBRT for brain metastasis, which showed failure of TKI treatment. Finally, we performed targeted deep sequencing to further analyze mutated genes in the cfDNA samples from NSCLC patients. We obtained sufficient cfDNA from six cases (1, 2, 12, 15, 16, and 17) after radiotherapy for sequencing and digital PCR analysis (>60 ng). The results are shown in Table 2. *EGFR* mutations were detected in post-radiotherapy samples from the four *EGFR* mutation-positive NSCLC patients. Only two out of four cases (case 15, case 17) showed these mutations in their pre-radiotherapy samples. Furthermore, we detected *EGFR* G719C mutation in the post-radiotherapy sample from patient (case 1), whose biopsy was not performed and the *EGFR* mutation status was unknown previously. In case 16, we detected the *EGFR* L747S mutation, which is known to confer resistance to TKIs. We also observed an increase in allele frequency and tumoral DNA content in 5 out of 6 cases (2, 12, 15, 16 and 17).

## DISCUSSION

Tumoral cfDNA analysis is useful for making cancer diagnoses and for assessing cancer-related drug resistance. However, this strategy is not viable in early stage cancer patients and those undergoing cancer treatments because the concentration of cfDNA is limited. In this study, therefore, we demonstrated that radiotherapy increases tumoral cfDNA levels in the plasma of stage I and IV NSCLC patients. We also demonstrated drug resistance-related *EGFR* gene mutations in the cfDNA of stage IV NSCLC patients that experience distal metastasis recurrence as a result of drug failure.

We subjected patients to radiotherapy (50 Gy/4 fr, 75 Gy/25 fr, 35 Gy/3 fr, or 23 Gy/1 fr) and analyzed total cfDNA levels in pre-RT, RT and post-RT plasma samples daily. We observed higher total and tumoral cfDNA levels at 24 h after irradiation than in pre-RT samples in stage I–II NSCLC patients. The total and tumoral cfDNA levels peaked at 7 days after irradiation. This suggested that cancer tissue apoptosis occurs within 24 h after radiotherapy and is similar to the kinetics of apoptosis in the cancer cell lines. Clinically, tumor reduction is observed at 4–8 weeks after radiotherapy as defined response evaluation criteria in solid tumours:Revised RECIST guideline (version 1.1). This large time lag between effects at a cellular level and at an anatomical level is probably due to radiation-induced inflammation and edema, which delays visual detection (e.g., by CT scan) of tumor reduction. We also observed elevated cfDNA levels after irradiation with a total dose of 30 Gy or less in 12 cases. Low irradiation doses of 8 Gy/1 fr to 30 Gy/10 fr are used for palliative treatment [28–30].

Radiation-induced apoptosis generates 50–300 kb fragments of DNA in cell lines [31]. However, cfDNA levels have not been determined in patients that have undergone radiotherapy. In this study, we quantified cfDNA levels after irradiation using digital PCR and amplicon sequencing. In a previous study, the status of drug resistance was monitored by determining the levels of fused cancer-related genes with >10 ng total cfDNA and allele frequency of 0.1% [16]. However, total cfDNA levels are limiting in early-stage cancer patients and those undergoing therapy. In our study, we obtained >10 ng cfDNA in 6 out of 11 stage I–II NSCLC patients that underwent radiotherapy. Moreover, we obtained >10 ng cfDNA in 9 out of 10 patients by increasing the whole blood volume to 14 mL, thereby augmenting allele frequency by about 10 fold. A previous report indicated that a depth of >10000× was required for cfDNA analysis in early cancer [19]. Moreover, increasing the allele frequency is cost-effective and extends the sequence (e.g., exome) analysis.

Radiotherapy is as effective as surgery for patients with stage I–II NSCLC [32]. However, surgery is preferred because it also provides specimens for comprehensive clinical diagnosis, especially in patients from whom biopsy specimens cannot be obtained. In some cases, patients undergoing radiation therapy experience functional decline in breathing and require anticoagulant medical treatment. Moreover, biopsy specimens can be obtained endoscopically in only 50–70% of cancer patients [33]. Detection of cancer-specific DNA mutations can diagnose secondary cancer and pulmonary metastasis in stage I–II patients that have undergone curative radiotherapy. This information is essential to make an informed decision regarding the next course of treatment. For example, follow-up CT is required every 3 months after SBRT. However, if benign nodule is confirmed, follow-up CT is required only once a year. Furthermore, distant metastasis recurrence is more prevalent in NSCLC patients with *EGFR* mutations than patients with wild-type *EGFR* [34, 35]. This information is useful for physicians and radiologists to follow-up cancer patients.

Molecular targeted therapy is preferred for stage IV patients with anaplastic lymphoma kinase (ALK) fusion or *EGFR* mutations. However, cancer cells with *EGFR* T790M and *ALK* L1196M mutations acquire drug resistance. The second-generation ALK repressor (Alectinib) for the *ALK* L1196M mutation and the third-generation drug for the *EGFR* T790M mutation are approved for some instances of resistance after TKI treatment [36, 37]. Screening of drug-resistant mutations by rebiopsy is difficult and hinders the use of new-generation molecular targeted therapies. Hence, liquid biopsy is the optimal choice in the future. However, cfDNA detection is difficult in treated patients. Tumor cannot be accurately genotyped if the patient presents several metastatic lesions.

In this study, we performed digital PCR and sequence analysis in four patients (cases 12, 15, 16, and 17) that were diagnosed with NSCLC bearing *EGFR* mutations and underwent TKI treatment. These patients received SRS with CyberKnife to treat brain metastasis as a result of treatment failure or emergence of drug resistance. In previous studies, cfDNA analysis showed increased drug-resistant mutations in the plasma of patients with a failed TKI treatment [38, 39]. The predominant drug-resistant mutation in NSCLC patients is *EGFR* T790M. Our study detected *EGFR* T790M mutation in two out of the four patients. Most patients with NSCLC are associated with brain metastasis and undergo radiotherapy with or without TKI treatment [40]. In patients treated with SRS for brain metastasis, active lesions are found only in the brain, and the number of metastases is limited to 4–10. Our study demonstrates that radiotherapy in such patients enables detection of tumor-associated gene mutations in the cfDNA samples.

When local failure occurs during systemic chemotherapy, radiotherapy is preferred for palliative care. Our study suggests that cfDNA detection combined with radiotherapy could replace rebiopsy to determine the tumor genotype.

Leon *et al.* and Cheg *et al.* evaluated total cfDNA levels using traditional irradiation methodology and suggested that irradiation induces cancer cell apoptosis, thereby increasing tumoral DNA in the plasma (Cancer Res. 1977 Mar; 37(3):646–50.) [41, 42]. In traditional radiotherapy, irradiation field includes the lymph region and the large blood vessel, which results in the apoptosis of lymphocytes in the blood vessels at a dose of around 2 Gy [43]. This increases total cfDNA, but hinders sequencing of tumor-derived DNA. We performed pinpoint irradiation which can avoid such hematopoietic cell bias (Supplementary Figure 1**)** the cancer tissue using stereotactic radiosurgery (SRS) and stereotactic radiotherapy (SRT) and confirmed tumoral cfDNA by digital PCR and sequence analysis. While our study speculates that the tumoral cfDNA was derived from apoptotic tumor cells, we confirm that irradiation increases tumoral cfDNA substantially. Importantly, we also clarified the time course of cfDNA elevation with clinical dose irradiation. The time course of apoptosis with irradiation is investigated previously in cell line and animal experiment, however the dose and fraction are clearly different as is described in introduction.

CfDNA analysis was approved by Food and Drug Administration (FDA) in 2017 and has been used clinically for diagnostic and prognostic applications. Our study investigated the influence of radiation on cfDNA quantity and composition.

Our study has several limitations. First, we performed digital PCR and targeted sequencing in a single experiment because of low recovery. Second, the sample size in our study was small. For that reason, we cannot completely exclude the possibility of technical error. However, to overcome clinical and biological bias, we selected only cases that were treated using pinpoint irradiation methods to avoid normal tissue contamination and chemotherapy effects. We also performed daily blood tests during radiotherapy and analyzed the digital PCR and targeted sequencing. We conclude that our results reflect tumor cfDNA kinetics during and after irradiation in the 17 NSCLC patients.

Our study demonstrates that radiotherapy increases total and tumoral cfDNA levels in early-stage and advanced stage NSCLC patients. Moreover, we demonstrate the utility of tumoral cfDNA analysis after irradiation for assessing drug-resistant mutations, which can help when preparing strategies for subsequent molecular targeted therapy.

## MATERIALS AND METHODS

### Study design and patients

The study protocols were carried out as approved by the Ethics committee of the Tokyo Metropolitan Cancer and Infectious Diseases Centre at Komagome Hospital (#2013-1233). We obtained written informed consent from all the study subjects for the use of blood and resected tumor tissue for research purposes. All samples and medical data used in this study were anonymous to protect patient information.

We enrolled 17 patients diagnosed with NSCLC between July 2013 and July 2015 at the Komagome hospital. The patients underwent curative or palliative radiotherapy. The main endpoint of this study was to evaluate cfDNA levels after radiotherapy. Tumors were diagnosed as NSCLC based on the histopathology of biopsy specimens and the patients were graded according to the classification of the Union for International Cancer Control 7th edition. Tumor genotyping was performed by PCR. The clinicopathological features of the patients are summarized in Table 1.

As shown in Figure 1, blood samples were obtained from all patients just prior to radiotherapy (pre-radiotherapy or pre-RT) or a day after the first radiotherapy session and a day after administration of 1/4, 1/2, and 3/4 of the total dose (during radiotherapy or RT) and at the end of the radiation regimen and at the first week and first month after radiotherapy (post-radiotherapy, post-RT). Among the seventeen enrolled patients that underwent radiotherapy, eleven were stage I–II NSCLC patients that rejected surgery and opted for radiotherapy. The remaining six stage IV NSCLC patients were diagnosed as *EGFR* mutation positive and presented with brain metastasis after TKI treatment failed.

### Radiotherapy

We used the computed tomography (CT) datasets for these patients, including fully delineated targets and organs-at-risk (OAR) to strategize subsequent radiotherapy. The treatment plans included a prescribed dose of 50 Gy/4 fr or 75 Gy/25 fr for primary stage I–II NSCLC patients and 23 Gy/1 fr or 35 Gy/3 fr to treat NSCLC patients with brain metastasis. We reduced the radiation dose to 45 Gy/4 fr in case 11 to avoid complications because of OAR. For stage IV disease, we performed only stereotactic radiosurgery (SRS) to the brain and did not use radiation therapy at other sites.

We prescribed 50 Gy/4 fr SBRT for NSCLC patients with negative lymph nodes of 3 cm or less and three-dimensional (3D) conformal radiotherapy (3D-CRT) at 75 Gy/25 fr for patients with 3–5 cm tumors in the primary site with adjacent risk organs. The doses were determined according to standard rules as is described in the National Comprehensive Cancer Network (NCCN) and Japan guideline. Stereotactic body irradiation therapy (SBRT) to the brain was performed using Cyber Knife (Accuray, Tokyo, Japan), and radiation treatment of the lungs was performed using Vero4DRT (Mitsubishi, Tokyo, Japan) (Supplementary Figure 1).

### Plasma collection

Whole blood was collected in 7-mL vacutainer tubes containing EDTA and centrifuged for 5 min at 800 × *g*. Cleared plasma was stored in cryostat tubes at –80° C until use. Plasma isolation was performed within 30 min of blood collection to prevent DNA contamination from blood cells.

### CfDNA extraction

We concentrated 1 mL patient plasma using Maxwell rapid sample concentrator (Promega, Madison, WI, USA) and extracted nucleic acids using the Maxwell blood purification kit (AS1480; Promega, Madison, WI, USA) according to the manufacturer’s instructions. DNA was purified using the QIAamp Circulating Nucleic Acid Kit (55114; Qiagen, Inc., Valencia, CA, USA) according to the manufacturer’s instructions. We eluted the DNA in 50 μL double distilled water and stored at –20° C. The DNA concentration was quantified with a Quantus fluorometer (Promega, Madison, WI, USA) following the manufacturer’s instructions.

### Digital quantitative PCR

Allele-specific qRT-PCR was performed in a LightCycler 480 Real-Time PCR System (Roche Holding AG., Basel, Switzerland). We determined the copy number of mutant and wild-type alleles of *EGFR*, *EGFR* exon 19 deletion as well as *EGFR* L858R and T790M mutations using a TaqMan SNP Genotyping kit (Thermo Fisher, Waltham, MA, USA) according to the manufacturer’s instructions (10 ng of cfDNA per reaction). Patient and standard samples were processed in duplicates, and the mean cycle threshold (Ct) value of duplicates was determined. The percentage of mutant *EGFR* alleles was calculated as the ratio of the copy number of mutant *EGFR* relative to the total copy number of *EGFR* (wild-type and mutant).

### CfDNA sequencing and analysis

For the sequencing assays, we used two commercial and validated panels: the Ion AmpliSeq Cancer Hotspot Panel v2 and the Ion AmpliSeq Colon and Lung Cancer Panel [26]. The Ion AmpliSeq Cancer Hotspot Panel v2 was designed to amplify 207 amplicons covering approximately 2800 COSMIC mutations from 50 oncogenes and tumor-suppressor genes, whereas, the Ion AmpliSeq Colon and Lung Cancer Panel evaluated 22 genes that are implicated in colon and lung cancers. We generated barcoded libraries using 3 ng each of cfDNA and the Ion AmpliSeq library preparation kit v2.0 (Life Technologies, Carlsbad, CA, USA). The samples were quantified using the Agilent 2100 Bioanalyser and Qubit 2.0 Fluorometer (Life Technologies), diluted to a final concentration of 10 pM for template preparation using the OneTouch 2 instrument and Ion One Touch Template kit v2 (Life Technologies, Carlsbad, CA, USA). The quality of the resulting pooled libraries was checked using the Ion Sphere quality control Kit in a Qubit 2.0 Fluorometer. Libraries that passed quality control tests were sequenced on a PGM Ion Torrent (Life Technologies) using the PGM 200 sequencing kit v2 and 318 Chip v2. We pooled 16 libraries to achieve 500× coverage per target amplicon.

We aligned FASTQs to the human genome (hg19), and identified point mutations using Torrent Suite Software v3.0 and the Ion Torrent Variant Caller v4.0 Plug-in using somatic high stringency parameters and the targeted and hotspot pipelines. We set a 5% allele frequency (AF) threshold and a 500× minimum coverage to identify de novo mutations; we also selected 0.1% AF to identify previously characterized mutations during treatment [27]. All identified variants were confirmed using IGV 2.3 (Broad Institute).

### Statistical analysis

We used Student’s *t*-test to compare cfDNA amounts at pretreatment versus the highest point under radiotherapy with EZR [44]and the final point as post-radiotherapy.

## SUPPLEMENTARY MATERIALS FIGURE


