# Correction: Radiotherapy increases plasma levels of tumoral cell-free DNA in non-small cell lung cancer patients

**DOI:** 10.18632/oncotarget.25378

**Published:** 2018-05-04

**Authors:** Shun-Ichiro Kageyama, Keiji Nihei, Katsuyuki Karasawa, Takeshi Sawada, Fumiaki Koizumi, Shigeo Yamaguchi, Shunsuke Kato, Hidehiro Hojo, Atsushi Motegi, Katsuya Tsuchihara, Tetsuo Akimoto

**Affiliations:** ^1^ Tokyo Metropolitan Cancer and Infectious Diseases Center Komagome Hospital, Bunkyo-ku, Tokyo 113-8677, Japan; ^2^ Juntendo University Main Hospital, Tokyo 113-8431, Japan; ^3^ National Cancer Center Hospital East, Kashiwa, Chiba 277-8577, Japan

**This article has been corrected:** The correct author names and affiliations are given below:

**Shun-Ichiro Kageyama^1,3^, Keiji Nihei^1^, Katsuyuki Karasawa^1^, Takeshi Sawada^1^, Fumiaki Koizumi^1^, Shigeo Yamaguchi^2^, Shunsuke Kato^2^, Hidehiro Hojo^3^, Atsushi Motegi^3^, Katsuya Tsuchihara^3^ and Tetsuo Akimoto^3^**

^1^ Tokyo Metropolitan Cancer and Infectious Diseases Center Komagome Hospital, Bunkyo-ku, Tokyo 113-8677, Japan

^2^ Juntendo University Main Hospital, Tokyo 113-8431, Japan

^3^ National Cancer Center Hospital East, Kashiwa, Chiba 277-8577, Japan

Original article: Oncotarget. 2018; 9:19368-19378. https://doi.org/10.18632/oncotarget.25053

