# Supplementary material for: Radiotherapy increases plasma levels of tumoral cell-free DNA in non-small cell lung cancer patients
Source: Oncotarget. 2018 Apr 10;9(27):19368–78. doi: 10.18632/oncotarget.25053 (PMC5922403; doi:10.18632/oncotarget.25053)
Supplement: Supplementary file 1 [file oncotarget-09-19368-s001.pdf]

Radiotherapy increases plasma levels of tumoral cell-free DNA in non-small cell lung cancer patients

SUPPLEMENTARY MATERIALS

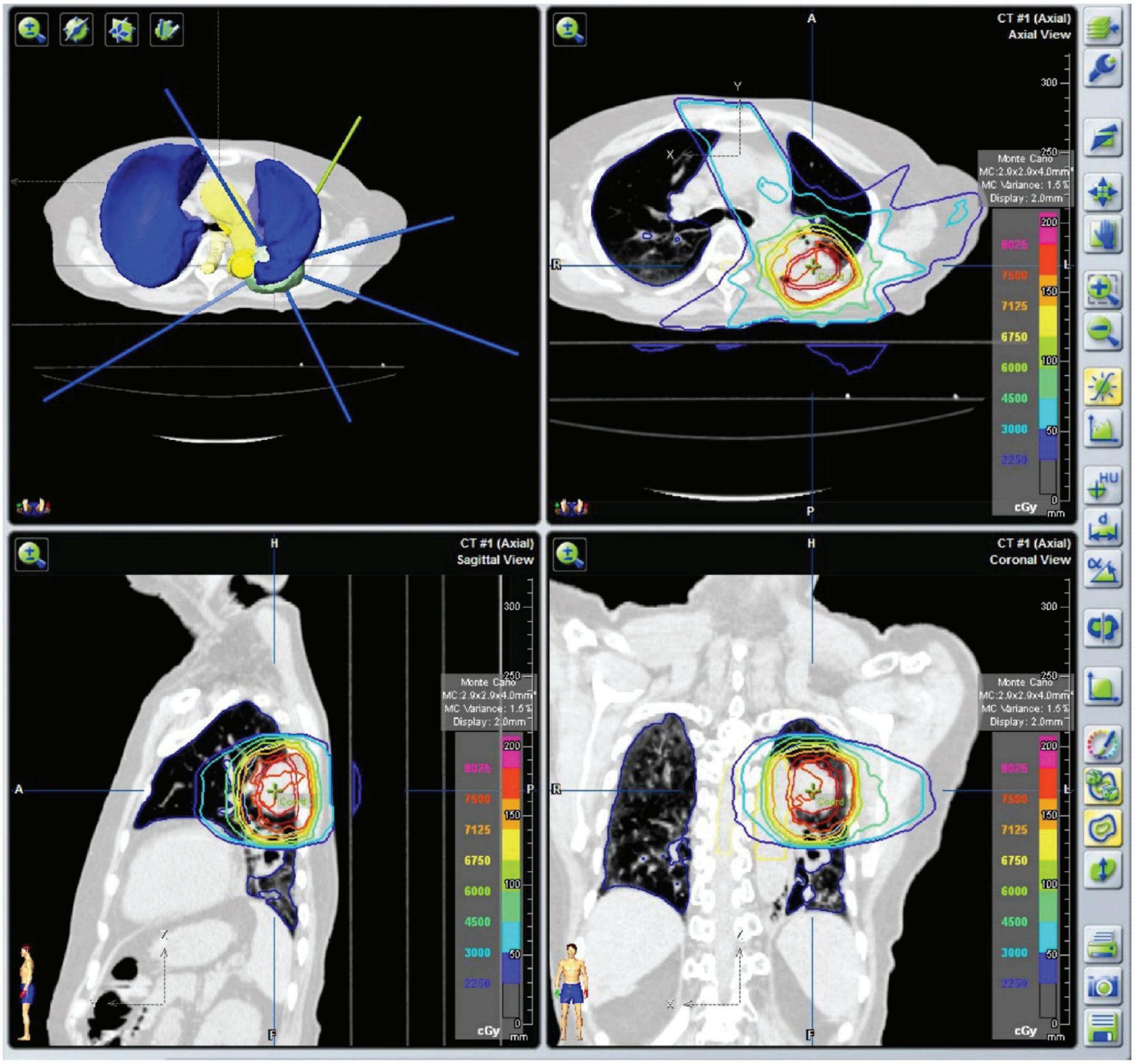

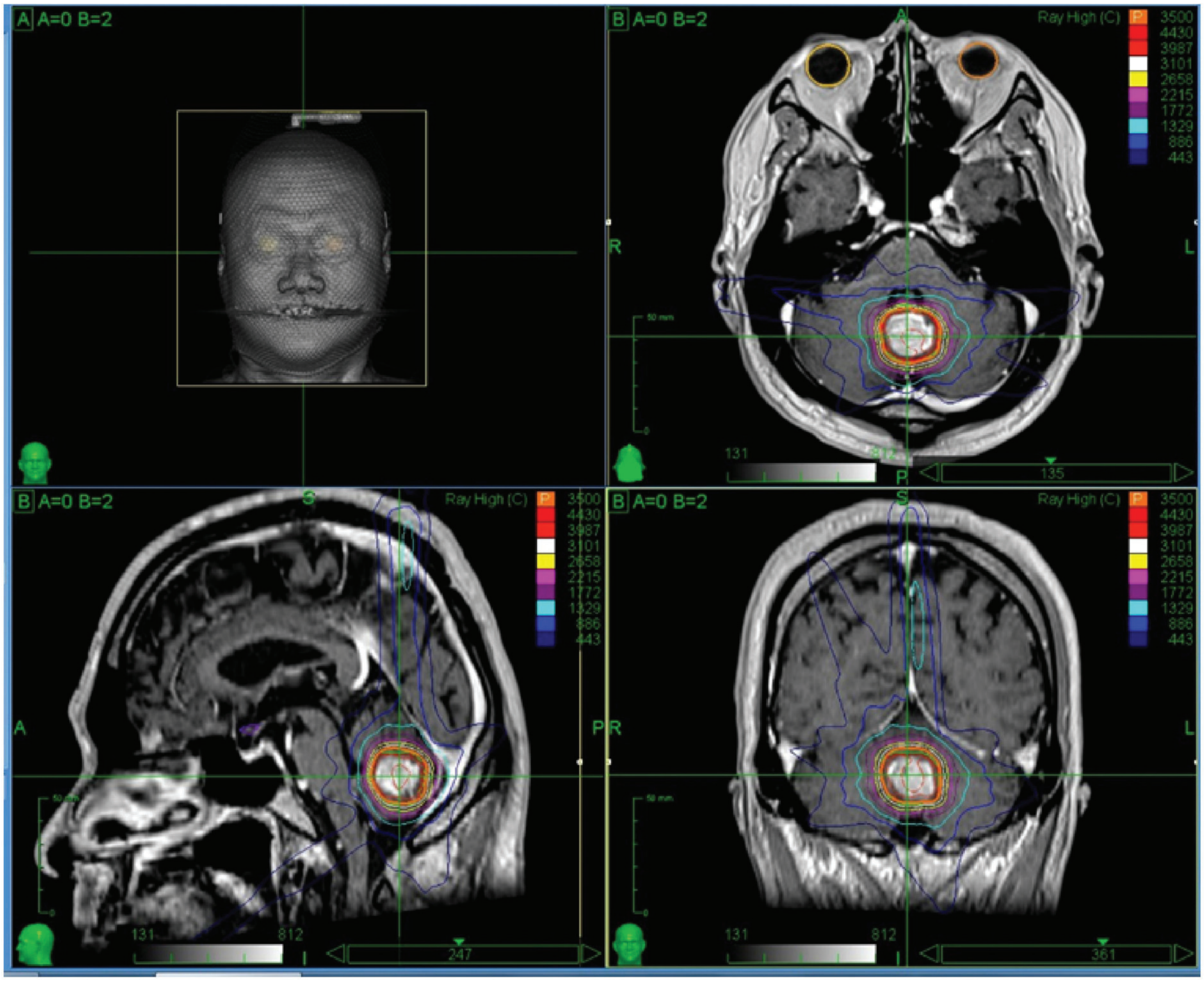

Supplementary Figure 1: Details of radiation treatment strategy. Representative CT images show the contoured growth tumor volume (GTV) in the brain and the lungs. Representative 4D-CT image shows the clinical target volume (CTV) for a margin of 3–5 mm, and an internal target volume (ITV). The evaluated planning target volume (Patel, defined as the PTV minus spinal cord) is estimated to receive the planned radiation doses in at least 95% of the volume (D95; Figure 1). The radiation treatment plan was prepared and evaluated using Brainlab iPlan (Munich, Germany) for irradiation to the lung and Accuray multiplan for brain metastasis. All contours and treatment plans were checked by two or more radio-oncologists.
